# The Impact of COVID-19 on the Prevalence and Perception of Telehealth Use in the Middle East and North Africa Region: Survey Study

**DOI:** 10.2196/34074

**Published:** 2023-02-02

**Authors:** Khalid Adnan Shamiyah, Simon Whitebridge, Nitya Kumar, Khaled Aljenaee, Stephen L Atkin, Khawla Fuad Ali

**Affiliations:** 1 Royal College of Surgeons in Ireland-Medical University of Bahrain Busaiteen Bahrain; 2 Al-Adan Hospital Kuwait City Kuwait

**Keywords:** COVID-19, telehealth, Instagram, WhatsApp, social media, telemedicine, impact, prevalence, perception, view, usage, utilization, safety, acceptability, survey

## Abstract

**Background:**

Due to the COVID-19 pandemic, telehealth has become a safer way to access health care. The telehealth industry has rapidly expanded over the last decade as a modality to provide patient-centered care. However, the prevalence of its use and patient acceptability remains unclear in the Middle East and North Africa (MENA) region.

**Objective:**

The primary aim was to assess the prevalence of telehealth use before and during the pandemic by using social media (Instagram) as an online platform for survey administration across different countries simultaneously. Our secondary aim was to assess the perceptions regarding telehealth among those using it.

**Methods:**

An Instagram account that reaches 130,000 subjects daily was used to administer a questionnaire that assessed the current prevalence of telehealth use and public attitudes and acceptability toward this modality of health care delivery during the COVID-19 pandemic.

**Results:**

A total of 1524 respondents participated in the survey (n=1356, 89% female; median age 31 years), of whom 97.6% (n=1487) lived in the Gulf Cooperation Council (GCC) region. Prior to COVID-19, 1350 (88.6%) had no exposure to telehealth. Following the COVID-19 pandemic, telehealth use increased by 251% to a total of 611 users (40% of all users). About 89% (571/640) of telehealth users used virtual visits for specialist visits. Of the 642 participants who reported using telehealth, 236 (36.8%) reported their willingness to continue using telehealth, 241 (37.5%) were unsure, and 164 (25.5%) did not wish to continue to use telehealth after the COVID-19 pandemic. An inverse trend, although not statistically significant, was seen between willingness to continue telehealth use and the number of medical comorbidities (odds ratio [OR] 0.81, 95% CI 0.64-1.03; *P*=.09). Compared to the respondents who chose only messaging as the modality they used for telehealth, respondents who chose both messaging and phone calls were significantly less likely to recommend telehealth (OR 0.42, 95% CI 0.22-0.80; *P*=.009). Overall, there was general satisfaction with telehealth, and respondents reported that telehealth consultations made them feel safer and saved both time and money.

**Conclusions:**

Telehealth use increased dramatically after the COVID-19 pandemic, and telehealth was found to be acceptable among some young adult groups on Instagram. However, further innovation is warranted to increase acceptability and willingness to continue telehealth use for the delivery of health care.

## Introduction

The worldwide pandemic caused by COVID-19 created a global challenge for health care systems. Data has shown that hospitalization was required in 20% of COVID-19 patients older than 80 years and varied from 0.04% to 16% in 10-year-olds and 70-year-olds, respectively [1]. The increasing demand and lack of sufficient facilities and resources have overwhelmed health care systems worldwide. With technical and staffing limitations, health care providers needed to employ new modalities to deliver appropriate medical care. As a result, telehealth technology has become a strategy for providing essential care when in-person consultations are limited.

Fueled by the abundance of internet-connected devices (63% of the global population have internet access), telehealth offered clinicians and patients an opportunity to use virtual communication to enhance access to medical care during the COVID-19 pandemic [2]. The definition of telehealth from the World Health Organization (WHO)’s *Recommendations on Digital Interventions for Health System Strengthening* is as follows: “The provision of healthcare services at a distance with communication conducted between healthcare providers seeking clinical guidance and support from other healthcare providers (provider-to-provider telehealth); or conducted between remote healthcare users seeking health services and healthcare providers (client-to-provider telehealth)” [3]. Various forms of telehealth services, including text messaging, email, patient portals, and video calls, have become increasingly available worldwide to cater to this newly created niche. However, before the COVID-19 pandemic was declared in March 2020, the adoption of telehealth into routine clinical practice had been slow due to the preference for face-to-face consultations. With the inevitable shift in the use of digital power to meet the needs and wants of today’s consumers, it has been suggested that telehealth will increasingly be adopted over the next decade [4-8]. Therefore, knowledge of user acceptance and perceived user value in comparison to other modes of health care delivery is critical to understanding the role of telehealth in the future of health care delivery and patient-doctor interactions.

Studies regarding telehealth in the Middle East and North Africa (MENA) region are scarce. Those available have limitations such as nonrepresentative sampling, assessment of a single form of telehealth, and the use of data originating from market research funded by organizations with a financial interest in presenting telehealth optimally [9]. To address this gap in the literature, we conducted a cross-sectional survey of telehealth use via a single social media platform (Instagram) in March 2021 to determine telehealth use and user-perceived value before and during the COVID-19 pandemic.

## Methods

### Study Population

The survey link was shared on the Instagram account of the principal investigator (KFA), which reaches 130,000 subjects daily. An Instagram post explaining our study’s scope and aims was shared, and an invitation to participants aged 18 years or older was extended. Followers of the account included subjects from all Arab countries, with the majority (>50%) residing in the Gulf Cooperation Council (GCC) region. Participants were allowed to fill out the survey in Arabic or English (Multimedia Appendix 1), the two predominantly spoken languages in the Middle East. Participants were allowed to access the survey link from March 17 to 29, 2021. The system we used ensured that participants were allowed to only complete the survey once. Resharing of the survey link was permitted to allow for snowball sampling.

### Survey Administration

The survey was split into 3 sections and 18 questions (detailed in Multimedia Appendix 1). The first section reflected respondent demographics, including questions about age, gender, education level, country of residence, and whether the respondent worked or studied in the health care sector. The second section identified the respondent’s engagement with the health care system and telehealth. If respondents answered yes to the use of telehealth, they were allowed to proceed to the third section of the survey, which was telehealth-specific and included questions on medical departments or specialties accessed, user satisfaction, the likelihood of continuing telehealth use, and the likelihood that a user would recommend it to others. The survey questions were designed based on preexisting surveys reported in the literature [10-12].

By clicking the web link provided on social media, participants were redirected to the online platform Zoho Creator (version 5.26; Zoho Corp), an online tool that simplifies conducting and analyzing surveys using custom and prebuilt templates. Before the commencement of the survey, participants were presented with a participant information sheet and a participant consent form. Data collected on Zoho were exported in a standard format and used for analysis.

### Data Collection and Analysis

Frequencies and percentages were used to describe the results obtained for categorical variables, while continuous variables were summarized as medians and IQRs. The distribution of telehealth users’ and nonusers’ covariates before and after the COVID-19 pandemic was used to determine predictors of telehealth use. Multivariable logistic regression was used to identify characteristics associated with the likelihood of continuing telehealth use and the respondent’s recommendation to others. The Pearson chi-square and Mann-Whitney tests were used to compare bivariate categorical and continuous variables, respectively. For model selection, we initially included all variables. Final model selection was informed by the parameters of goodness of fit, calibration, and discrimination. Analyses were conducted in Stata (version 17; StataCorp). The threshold for statistical significance was *P*<.05.

### Patient and Public Involvement

Survey respondents were not involved in designing the study; however, they played a crucial role in recruitment. As stated earlier, participants were permitted to reshare the survey link through social platforms, allowing for propagation of the survey’s spread via the snowball effect. Following publication, the results of this study will be shared with the public by producing an interactive video that will be disseminated on the initially used social media platform.

### Ethical Considerations

This study was designed and executed in accordance with the ethical guidelines of the Royal College of Surgeons in Ireland–Medical University of Bahrain. A complete application for ethical approval for human subject research was submitted to the Research Ethics Committee of the institution and approval was granted for the current study on January 18, 2021. The approval was granted for the primary data collection and analysis methods of this study. All data collected for the study were anonymous, with no personal identifiers collected from participants. No compensation was provided to the human subjects who participated in the study.

## Results

### Demographics

A total of 1524 respondents participated in the survey. Women comprised most of the respondents, with 1356 (89%) responses. The median age of the respondents was 31 years, with an IQR of 25 to 38 years. Overall, 1266 (83.1%) of the participants had a college degree or higher, 1487 (97.6%) lived in the GCC region, and 723 (47.4%) resided in large cities. Additional baseline characteristics are shown in Table 1.

**Table 1:** Respondents’ demographics and characteristics (N=1524).

Characteristic		Values	
Age (years), median (IQR)		31 (25-38)	
Female gender, n (%)		1356 (88.9)	
**Education level, n (%)**			
	Less than high school		28 (1.8)
	High school		230 (15.1)
	College		1113 (73)
	Postgraduate		153 (10)
**Region, n (%)**			
	Gulf Cooperation Council region		1487 (97.6)
	North Africa		37 (2.4)
**Living area, n (%)**			
	Rural		31 (2)
	Small town		515 (33.8)
	Suburb		255 (16.7)
	Large city		723 (47.4)
**Work or study in health care, n (%)**			
	Yes		280 (18.4)
	No		1244 (81.6)
**Number of current medical conditions, n (%)**			
	None		649 (42.6)
	1		653 (42.8)
	2		148 (9.7)
	3		47 (3.1)
	4 or more		27 (1.8)
**Number of clinic visits, n (%)**			
	None		175 (11.5)
	1 to 2		565 (37.1)
	3 to 4		438 (28.7)
	5 or more		346 (22.7)
**Number of pills, n (%)**			
	None		635 (41.7)
	1 to 2		652 (42.8)
	3 to 4		177 (11.6)
	5 or more		60 (3.9)
**Type of virtual visit (n=642), n (%)**			
	Message		177 (27.6)
	Message and phone call		322 (50.2)
	Message, phone call, and video		143 (22.3)
**First consultation (n=641), n (%)**			
	Yes (only first consultation)		215 (33.5)
	No (first consultation or follow-up and other visits)		426 (66.5)
**Primary or specialty care (n=640), n (%)**			
	Primary care visits only		69 (10.8)
	Specialist visits only or specialist visit and primary care visit		571 (89.2)
**Satisfaction with telehealth, n (%)**			
	Feels telehealth saves time (n=382)		215 (56.3)
	Feels telehealth saves money (n=380)		307 (80.8)
	Feels safe with telehealth (n=429)		283 (66)
	Feels telehealth respects privacy (n=264)		233 (88.3)
**Willing to continue using telehealth (n=641), n (%)**			
	Yes		236 (36.8)
	No		164 (25.6)
	Unsure		241 (37.6)
**Willing to recommend telehealth (n=641), n (%)**			
	Yes		385 (60.1)
	No		82 (12.8)
	Unsure		174 (27.1)

### Telehealth Use Before the COVID-19 Pandemic

Before the COVID-19 pandemic, 1350 of 1524 (88.6%) respondents did not have any telehealth modality exposure (video, phone, messaging, or other). The distribution of sociodemographic characteristics seemed to be fairly similar across the groups, as shown in Table 2. Telehealth use was found to be similar across education categories (*P*=.08) and did not differ significantly between those who studied or worked in health care and those who did not (*P*=.10).

**Table 2:** Determinants of telehealth use before COVID-19. *P* values are from the chi-square test unless otherwise noted.

Characteristic		Nonusers (n=1350)	Users (n=174)	*P* value
Age, median (IQR)		31.0 (25.0-38.0)	32.0 (26.0-39.0)	.11^a^
Female, n (%)		1200 (88.9)	156 (89.7)	.76
**Level of education, n (%)**				.08
	Less than high school	24 (1.8)	4 (2.3)	
	High school	214 (15.9)	16 (9.2)	
	College	982 (72.7)	131 (75.3)	
	Postgraduate	130 (9.6)	23 (13.2)	
**Country, n (%)**				.52
	Gulf Cooperation Council region	1316 (97.5)	171 (98.3)	
	North Africa	34 (2.5)	3 (1.7)	
**Living area, n (%)**				.17
	Rural	30 (2.2)	1 (0.6)	
	Small town	458 (33.9)	57 (32.8)	
	Suburb	232 (17.2)	23 (13.2)	
	Large city	630 (46.7)	93 (53.4)	
Work or study in health care		256 (19)	24 (13.8)	.10
**Number of current medical conditions, n (%)**				.11
	None	590 (43.7)	59 (33.9)	
	1	572 (42.4)	81 (46.6)	
	2	125 (9.3)	23 (13.2)	
	3	40 (3)	7 (4)	
	4 or more	23 (1.7)	4 (2.3)	
**Number of clinical visits, n (%)**				.51
	None	160 (11.9)	15 (8.6)	
	1 to 2	500 (37)	65 (37.4)	
	3 to 4	389 (28.8)	49 (28.2)	
	5 or more	301 (22.3)	45 (25.9)	
**Number of pills, n (%)**				.11
	None	572 (42.4)	63 (36.2)	
	1 to 2	573 (42.4)	79 (45.4)	

^a^Mann-Whitney test.

### Telehealth Use During the COVID-19 Pandemic

When comparing those who used telehealth upon the commencement of the COVID-19 pandemic versus those who did not (Table 3), we found that users had a significantly higher median age compared to nonusers (32.0 years vs 30.0 years; *P*=.001). Use also differed significantly across the categories of education (*P*=.01), existing medical conditions (*P*<.001), the number of pills taken (*P*<.001), and the number of clinic visits (*P*<.001). A significantly higher proportion of nonusers (20.6%) was found to comprise those who worked or studied in health care compared to users (15.1%, *P*=.006).

**Table 3:** Determinants of telehealth use after COVID-19. *P* values are from the chi-square test unless otherwise noted.

Characteristic			Nonusers (n=913)		Users (n=611)		*P* value	
Age (years), median (IQR)			30.0 (24.0-38.0)		32.0 (26.0-39.0)		.001^a^	
Female gender, n (%)			808 (88.5)		548 (89.7)		.47	
**Level of education, n (%)**								.01
	Less than high school	16 (1.8)		12 (2)				
	High school	151 (16.5)		79 (12.9)				
	College	671 (73.5)		442 (72.3)				
	Postgraduate	75 (8.2)		78 (12.8)				
**Country, n (%)**								.19
	Gulf Cooperation Council region	887 (97.2)		600 (98.2)				
	North Africa	26 (2.8)		11 (1.8)				
**Area of living, n (%)**								.63
	Rural	21 (2.3)		10 (1.6)				
	Small town	307 (33.6)		208 (34)				
	Suburb	159 (17.4)		96 (15.7)				
	Large city	426 (46.7)		297 (48.6)				
Work or study in health care			188 (20.6)		92 (15.1)		.006	
**Number of current medical conditions, n (%)**								<.001
	None	449 (49.2)		200 (32.7)				
	1	358 (39.2)		295 (48.3)				
	2	77 (8.4)		71 (11.6)				
	3	21 (2.3)		26 (4.3)				
	4 or more	8 (0.9)		19 (3.1)				
**Number of clinic visits, n (%)**								<.001
	None	148 (16.2)		27 (4.4)				
	1 to 2	371 (40.6)		194 (31.8)				
	3 to 4	249 (27.3)		189 (30.9)				
	5 or more	145 (15.9)		201 (32.9)				
**Number of pills, n (%)**								<.001
	None	440 (48.2)		195 (31.9)				
	1 to 2	361 (39.5)		291 (47.6)				
	3 to 4	94 (10.3)		83 (13.6)				
	5 or more	18 (2)		42 (6.9)				

^a^Mann-Whitney test.

### Increase in Telehealth Use Since Pandemic Onset

Within one year of the COVID-19 pandemic, the use of telehealth services increased by 251%, from 174 users (11.4% of all pre–COVID-19 users) of 1524 respondents before the advent of COVID-19 to 611 users (40% of all users since COVID-19) of 1524 respondents since the onset of COVID-19.

### Purpose of Virtual Visits and Specialties Accessed

About a third of the participants reported using telehealth for first-time consultations (33.5%, 215/641); the rest (66.5%, 426/641) reported using telehealth for follow-up visits and preoperative or postoperative visits. Of the respondents that reported using telehealth, 10.8% (69/640) used it to access primary care, and 89.2% (571/640) used it to access specialist visits (Table 1).

### Telehealth Modalities Used

Telehealth modalities used by the participants (Table 1) included only messaging (27.6%, 177/642), a combination of messaging and audio calls (50.2%, 322/642), and a combination of messaging, audio, and video calls (22.3%, 143/642). We did not find any participants who used only video calls but not messaging or audio or only audio calls but not messaging or video. Respondents who reported the use of calls were significantly less willing to continue using telehealth than those who reported the use of messaging (OR 0.58, 95% CI 0.35-0.96; *P*=.038; Table 4).

**Table 4:** Association between willingness to continue virtual visits and participant characteristics (N=400).

Characteristic			Odds ratio for willingness vs unwillingness to continue virtual visits (95% CI)		*P* value
Pre–COVID-19 telehealth user			1.73 (1.07-2.82)		.03
Post–COVID-19 telehealth user			1.40 (0.46-4.29)		.54
Age			1.01 (0.99-1.03)		.16
Number of existing medical conditions			0.81 (0.64-1.03)		.09
Works or studies in health care or allied field			1.09 (0.61-1.93)		.77
**Type of virtual visit**					
	Message	Reference category			
	Message and phone call	0.58 (0.35-0.96)		.04	
	Message, phone call, and video	1.00 (0.54-1.86)		.98	

### Respondents’ Satisfaction with Telehealth

More than half of the respondents felt that their privacy and confidentiality were respected when using telehealth (233/264, 88.3%) and that telehealth helped them feel safer (283/429, 66%). The majority of the telehealth users felt that telehealth saved money (307/380, 80.8%) and time (215/382, 56.3%; Table 1).

### Willingness to Continue Virtual Visits

Those who reported using at least one telehealth modality (n=641) were further asked about their willingness to continue their telehealth use following the COVID-19 pandemic. Of these, 236 (36.8%) reported they were willing to continue using telehealth, 241 (37.6%) were unsure, and 164 (25.6%) did not wish to continue to use telehealth after the COVID-19 pandemic (Table 1).

Among the 400 participants who reported being either willing or unwilling to continue using telehealth, we looked at the association between willingness to continue virtual visits and participant characteristics using a logistic regression model (Table 4). Respondents who used telehealth services before the COVID-19 pandemic were significantly more likely to continue using them than those without prior use of telehealth (OR 1.73, 95% CI 1.07-2.82; *P*=.03). An inverse trend between willingness to continue telehealth use and an increasing number of medical comorbidities was seen, although this was not significant (OR 0.81, 95% CI 0.64-1.03; *P*=.09). However, sicker patients (with a higher number of comorbidities) were found to be more likely to use telehealth after the onset of the pandemic (*P*<.001), as seen in Table 3.

### Willingness to Recommend Telehealth

We looked at the association between willingness to recommend virtual visits and participant characteristics (Table 5). Compared to the respondents who chose only messaging as the modality they used for telehealth, respondents who chose both messaging and phone calls had less than half the odds of recommending virtual visits (OR 0.42, 95% CI 0.22-0.80; *P*=.009). Respondents who reported taking 4 or more pills per day had significantly lower odds of recommending telehealth than those who took less than 4 pills daily (OR 0.30, 95% CI 0.09-0.98; *P*=.048). Age, existing medical conditions, and employment in a health-related field were not found to be associated with the willingness to recommend telehealth use.

**Table 5:** Association between willingness to recommend virtual visits and participant characteristics (N=466).

Characteristic		Odds ratio for willingness vs unwillingness to recommend virtual visits (95% CI)	*P* value
Pre–COVID-19 telehealth user		1.53 (0.80-2.92)	.19
Post–COVID-19 telehealth user		1.44 (0.40-5.12)	.57
Age		1.000 (0.97-1.02)	.96
Number of existing medical conditions		1.09 (0.76-1.55)	.62
Works or studies in health care or allied field		0.90 (0.44-1.85)	.79
**Type of virtual visit**			
	Message	Reference category	
	Message and phone call	0.42 (0.22-0.80)	.01
	Message, phone call, and video	0.90 (0.40-2.03)	.81
**Number of pills**			
	None	Reference category	
	1 to 2	0.57 (0.30-1.08)	.09
	3 to 4	1.47 (0.48-4.50)	.49
	4 or more	0.30 (0.09-0.98)	.048

## Discussion

### Principal Findings

The emergence of the COVID-19 pandemic swamped health care systems worldwide, necessitating the introduction of telehealth services to allow for remote health care delivery. Though many might postulate that telehealth is a temporary fix for a current global problem, projections expect telehealth to become a US $186.6 billion industry by 2026 [13]. Overall, our results highlighted the dramatic increase in telehealth use, its general acceptance among predominantly young, female respondents on social media, and its significant role in bridging barriers in health care delivery.

The IQR for the age of the respondents (24 to 38 years) showed that they tended to be on the younger end of the spectrum; this IQR was narrower than what is found in the literature [14,15]. Respondents in their mid to late thirties were more engaged with telehealth services than younger respondents in their early to mid-twenties before and after the COVID-19 pandemic. We hypothesize that this pattern was due to the platform (Instagram) our survey was conducted on. According to Statista, a company specializing in market and consumer data, as of April 2022, 30.2% of users on Instagram were women between the ages of 18 to 24 years, and over two-thirds of users on Instagram were aged 34 years or younger [16]. These numbers conform with the age range of our respondents. Furthermore, according to the literature, younger patients are more likely to access the internet and use smartphones, which are both necessities to access and interact with telehealth services, than older patients [2]. Even though a solid conclusion cannot be drawn due to the lack of representation of older age groups in our study, the literature reports up to 66% of telehealth consumers are between the ages of 18 and 49, and the remainder are older than 50 years [15,17].

In this study, 97.6% (1487/1524) of respondents were residents of countries within the GCC area. Although most countries in the MENA region share common cultural norms, language, and religion, their quality of life, access to health care, education, and financial resources vary substantially. On one end of the spectrum lie the resource-rich countries of the GCC region, and on the other are countries with ongoing political conflicts, famine, and poverty. A regional study conducted by Ram [18] showed that GCC countries have made significant investments in building infrastructure for an electronically centered health care system consisting of electronic health records, online medical education, and information security over recent years. In contrast, countries like Yemen, Syria, Palestine, and Iraq were less able to build more robust health care systems that could bear the technological weight imposed by telehealth and further advancements.

On a more global scale, the MENA region’s telehealth use before and after the pandemic was underwhelming compared to worldwide figures. A study published by the WHO in which 125 countries were surveyed to investigate the growth of telehealth further emphasized the lag experienced by countries within the MENA region when it came to telehealth implementation compared to European and North American countries [19]. These findings highlight the discrepancy in the level of technological readiness and penetration of telehealth within health care systems in countries in the MENA region during the COVID-19 pandemic [20].

The various types of telehealth delivery methods are usually classified as synchronous or asynchronous. Synchronous modalities allow for real-time user interaction, including video chat and telephone calls. Our study shows that video calls were more accepted among participants than telephone calls. We hypothesize that patients might have found it more challenging to describe their symptoms to their physicians over a telephone call. With telephone calls being generally less personal and more remote than video calls, patients usually struggle with communicating their symptoms over audio calls. Research has shown that patients tend to find it challenging to cultivate a therapeutic relationship without the aid of visual and nonverbal communication cues [21]. A previous study also supports this conclusion, as it showed that patients find difficulty in describing pain as a phenomenon to their health care providers, and they therefore feel misunderstood [22]. This is supported by a previous study that showed that pain can be a challenging phenomenon to describe for patients. The deficiency in good patient-physician relationships is undoubtedly an issue highlighted in the literature as one of the most critical influencers of patient satisfaction levels [23,24]. Video calls, on the other hand, can facilitate the exchange of both verbal and nonverbal cues, thereby allowing for the cultivation of a therapeutic relationship yielding higher patient satisfaction levels.

In contrast, asynchronous modalities allow for communication between users by storing and forwarding information over time without both parties needing to be present simultaneously. Essential forms include text messaging and image sharing [25]. Our results indicate that messaging was more acceptable than telephone calls among respondents. This result might be driven by the mobile phone revolution, specifically among younger generations. Given that most of the users on Instagram and survey respondents were young women, this finding was not surprising. The widespread use of multiple platforms like WhatsApp and iMessage has allowed physicians opportunities to interact in ways that are the most comfortable and familiar to patients. It has been found that when using a more comfortable communication medium, patients are more likely and willing to share sensitive information than in face-to-face encounters [26].

Our results showed that 11.4% (174/1524) of the respondents used telehealth before the pandemic, while considerably more respondents accessed telehealth services after the emergence of COVID-19 (40%, 611/1524), representing a 251% increase within one year of the pandemic. A similar trend in telehealth use has been reported in similar global studies [17]. Two possible factors may explain this sharp rise in telehealth use. First, to minimize the spread and exposure of infectious diseases, access to hospitals and clinics was limited, and a broader implementation of telehealth services was established. Second, the nationwide implementation of lockdowns and quarantines across the MENA region forced patients seeking medical attention to resort to a safer and more convenient way to access care via telehealth during the COVID-19 pandemic. Our results reflect this phenomenon: 66% (283/429) of respondents reported telehealth made them feel safer and more protected during the pandemic.

In assessing the permanency of telehealth use, we asked respondents about their willingness to continue its use in a postpandemic world. We found that 36.8% (236/641) of respondents intended to continue using telehealth services after the COVID-19 pandemic. While this figure may indicate a progressive future for telehealth, it was found to be significantly less than the value (66%) reported in the literature [27]. We postulate that one of the reasons for the discrepancy between our data and the literature may be due to the respondents’ lack of telehealth awareness. Our data also showed that those who declared the use of telehealth before the COVID-19 pandemic were almost twice as likely to continue their use compared to those who had no prior use of telehealth. These findings may suggest that those who had the chance to experiment with telehealth and make subjective judgments about such services were more accepting of the technology. As telehealth is in its adolescence, raising awareness and intensifying informative advertising may help equip respondents to make more informed judgments on telehealth.

Using the number of medical conditions a participant has as a proxy for their overall health status, our results showed a statistically significant relationship between the health status of participants and their telehealth use after the COVID-19 pandemic. This finding suggests that those with more medical comorbidities, who are most at risk of adverse health outcomes due to COVID-19, were more likely to participate in telehealth use. We hypothesize that this may be due to health care providers and more medically vulnerable participants opting for telehealth visits to avoid unnecessary travel and exposure to COVID-19.

Although not statistically significant, an inverse trend between respondents’ medical vulnerability and the likelihood of continuing telehealth use was found in our sample. This opposes data found in the literature, which shows that patients with increasing comorbidities tend to be more willing to continue engaging with telehealth [27]. However, it is worth noting that our study reflects only experiences among telehealth users, rather than the acceptability of telehealth by the general population. This limits our ability to make conclusive claims about willingness to continue using telehealth among our respondents.

While one cannot know what a postpandemic world will look like, one can postulate from trends in our study and the literature that telehealth is evolving and will continue to do so. However, the delay in telehealth integration within health care systems in the MENA region is perhaps due to the lack of innovation and the negligible amount of research conducted in the digital health field. To meet the increasing demand for telehealth within the region, a scale-up of telehealth capabilities and informational technology infrastructures is needed. Countries within the region should also start encouraging and investing in research initiatives to better understand patient preferences, the cost-effectiveness of such systems, and legal frameworks and policies [9].

Despite the high rates of telehealth adoption in the literature and uptake in our study, there is still skepticism about whether implementation was due to necessity or efficacy. It is essential to understand that telehealth use is not yet suitable for all patients, as a digital divide has been well documented in the literature among specific populations. Older patients, racial minority groups, and those from lower socioeconomic classes have shown lower rates of access to technology and the internet [28,29]. These dividers are more prominent in older populations, who have lived mainly without using such technologies [30]. Overcoming these hurdles can prove to be an extremely difficult task. In this light, telehealth can play a counterproductive role and may increase the health care disparity. Will the gap in technological literacy, access, and engagement of minority groups leave them at a disparity as health care becomes more technologically advanced and demanding? More inclusive studies are warranted to provide more conclusive samples, with uniform representation of these minority groups, before strong conclusions can be reached.

### Limitations

Our study had several limitations. Most important and perhaps most significant is our study’s survey design. When completing the survey, only those who answered yes to having used telehealth were asked about their perceptions and perspectives on telehealth. In retrospect, respondents who reported not using telehealth should have been asked for a reason for their nonuse and about their perceptions and perspectives on telehealth. Due to this design error, we cannot make general statements about the acceptance of telehealth among the general population, but only among those that have tried telehealth.

Due to demographic differences in the characteristics of those who inhabit social media, our sample was skewed toward women in younger age groups and could not help us understand older populations. This was perhaps also due to the content posted by the principal investigator on her social media page, which targeted conditions predominantly affecting younger women.

While it was a strength that the sample was concentrated within the GCC countries, the sample also lacked diversity within the greater MENA region. For future projects, a more inclusive sample of the MENA region is required to make more robust conclusions about the different patterns of telehealth use between countries within the region.

As with the majority of internet surveys, data integrity is of concern. Respondents may have interacted with the survey in a suboptimal way, such as by rushing through and picking random answers. Such behavior can lead to undesirable responses that may have affected the results [31]. Finally, due to our limited sample size, many associations were not statistically significant, potentially due to the study’s low power.

### Conclusions

The COVID-19 pandemic has transformed the delivery of health care around the world. Since the start of the pandemic, our study has shown that even among only young women, there has been a dramatic 251% increase in telehealth use within the MENA region. Participants reported that telehealth made them feel safer and was more efficient than conventional visits. It is unclear whether future telehealth use trends will continue to rise, especially if temporary policies to facilitate telehealth services are retired. Public health officials and surveillance systems can use this data to fund and promote further essential innovation in telehealth to allow for the maintenance of telehealth use trends within the region. Regardless of whether health care systems are ready for such a shift, immediate action is warranted to adapt to virtual care.

## Appendix

Multimedia Appendix 1 Consists of (1) survey in English and Arabic, (2) participation information form in English and Arabic, and (3) participant consent form in English and Arabic.

## Abbreviations

GCC: Gulf Cooperation Council
MENA: Middle East and North Africa
OR: odds ratio
WHO: World Health Organization
