# Supplementary material for: The Impact of COVID-19 on the Prevalence and Perception of Telehealth Use in the Middle East and North Africa Region: Survey Study
Source: JMIR Form Res. 2023 Feb 2;7:e34074. doi: 10.2196/34074 (PMC9897307; doi:10.2196/34074)
Supplement: Multimedia Appendix 1 [file formative_v7i1e34074_app1.docx]

# **Appendix 1**

## Survey (English)

1. Gender (F/M)
2. Age (state)
3. Education level (highest)
   - - less than high school
     - high school
     - college/university
     - Post-graduate studies
4. Current medical problems (select all that applies)

- Diabetes
- High blood pressure
- High blood cholesterol
- Thyroid disease
- Arthritis
- Heart disease
- Lung disease
- Cancer (current or in remission)
- Others:

1. Country of current residence (select one) – drop down menu of all countries
2. Do you work or study in the healthcare sector? (yes/no)
3. Please characterize your current living community:
   - - Large city
     - Small city or town
     - Suburb near a large city
     - Rural area
4. On average, how often do you visit healthcare provider(s) for your health in a year?
   - - Never
     - 1-2 times
     - 3-4 times
     - 5 or more times
5. On average, how many kinds of pills do you take in a day?
   - I do not take any
   - 1-2 types
   - 3-4 types
   - 5 or more types
6. Have you ever had an appointment with a doctor, nurse, or any other health professional virtually by video call or by phone call **before** the COVID-19 pandemic? (yes/no)
7. Have you ever had an appointment with a doctor, nurse, or any other health professional virtually by video call or by phone call **since the start** of the COVID-19 pandemic? (yes/no)

If you answered {Yes} to questions 10 and/or 11, please proceed with question 12.

If you have answered {No} to both questions 10 AND 11, this ends the survey for you. Thank you for your participation. You may wish to contact us at [covidstudybh@rcsi.com](mailto:covidstudybh@rcsi.com) if you have any questions or concerns.

1. My virtual visit(s) was(ere) through: (select all that applies)

- Phone call(s)
- Video call(s)
- Message chat(s)
- Other:

1. What was the purpose of your virtual appointment(s)? (select all that applies)

- First time consultation(s)
- Follow-up visit(s)
- Pre-operative visit(s)
- Post-operative visit(s)
- Other:

1. Which department(s)/specialtie(s) was your virtual appointment with? (select all that applies)

- Primary care
- Surgery
- OB/GYN
- Dermatology
- Psychiatry/Psychology
- Endocrinology
- Gastroenterology
- Hematology/Oncology
- Nephrology
- Cardiology
- Pulmonology
- Others:

1. Compared to in-office visits, virtual visits were more effective/equally effective/less effective in: (((REC: this will be in table format)))

- Saving time (more effective/equally effective/less effective)
- Saving money/expenses (more effective/equally effective/less effective)
- Conversing with your healthcare provider (more effective/equally effective/less effective)
- Addressing my questions/inquiries (more effective/equally effective/less effective)
- Satisfying my overall healthcare needs (more effective/equally effective/less effective)
- Feeling safer to have the telephone conversation than visit the doctor because COVID-19 disease (more effective/equally effective/less effective)
- Respecting my privacy and confidentiality (more effective/equally effective/less effective)

If a medication(s) was suggested/prescribed, how did you pick it up?

Pharmacy pick up

Home delivery of medication

I was not prescribed a medication

1. Would you continue to use virtual appointments after the COVID-19 pandemic is over? (yes/no/not sure)
2. Would you recommend virtual visits to others? (yes/no/not sure)

### Survey (Arabic)

 ١. الجنس (أنثى / ذكر)

٢. العمر (اذكر)

٣.  مستوى التعليم (الأعلى)

- أقل من الثانوية
- العامة
- الكلية/الجامعة
- الدراسات العليا

٤. المشكلات الطبية الحالية (حدد كل ما ينطبق)

- مرض السكري
- ارتفاع ضغط الدم
- ارتفاع كلسترول الدم
- أمراض الغدة الدرقية
- التهاب المفاصل
- أمراض القلب
- أمراض الرئة
- أورام
- أخرى:

٥. بلد الإقامة الحالية (اختر واحدة)

٦. هل تعمل أو تدرس في قطاع الرعاية الصحية؟ (نعم / لا)

٧. يرجى وصف مجتمعك السكني الحالي:

- مدينة كبيرة
- مدينة صغيرة أو بلدة
- ضاحية بالقرب من مدينة كبيرة
- المنطقة الريفية

٨. تقريبا​​، كم مرة تزور مقدم (مقدمي) الرعاية الصحية من أجل صحتك خلال عام؟

- بدا
- ١-٢ مرات
- ٣-٤ مرات
- ٥ أو أكثر

٩. تقريبا​​​​، كم عدد أنواع الحبوب التي تتناولها في اليوم؟

- أنا لا آخذ أي
- ١-٢ نوع
- ٣-٤ أنواع
- ٥ أنواع أو أكثر

١٠. هل سبق وان كان لك موعدًا افتراضيا مع طبيب أو ممرضة أو أي متخصص صحي آخر عن طريق مكالمة فيديو أو عبر مكالمة هاتفية قبل أن حدث وباء COVID-19؟ (نعم / لا)

١١.  هل سبق وان كان لك موعدًا افتراضيا مع طبيب أو ممرضة أو أي متخصص صحي آخر عن طريق مكالمة فيديو أو مكالمة هاتفية منذ بداية أن حدث وباء COVID-19؟ (نعم / لا)

إذا أجبت ب"لا" لكلا السؤالين 10 و 11، فقد انتهيت من الإجابة على الاستبيان. نشكرك على مشاركتك القيمة. لأي استفسار الرجاء ابلاغنا على البريد الالكتروني التالي: [covidstudybh@rcsi.com](mailto:covidstudybh@rcsi.com). اذا اجبت ب"نعم" لأي من السؤالين 10 أو 11 أو كلاهما، فالرجاء المواصلة بالإجابة على الأسئلة أدناه:

١٢. كانت زيارتي (زياراتي) الافتراضية من خلال: (حدد كل ما ينطبق)

- المكالمات الهاتفية
- مكالمة (مكالمات) الفيديو
- محادثة (محادثات) الرسائل
- اخر:

١٣.  ما هو الغرض من موعدك (مواعيدك) الافتراضي؟ (حدد كل ما ينطبق)

- استشارة (استشارات) المرة الأولى
- متابعة زيارة
- زيارة (زيارات) ما قبل الجراحة
- زيارة ما بعد العمليات الجراحية
- اخر:

١٤.  ما القسم (الأقسام) / التخصص (التخصصات) الذي كان موعدك الافتراضي معه؟ (حدد كل ما ينطبق)

- الرعاية الأولية
- الجراحة
- أمراض النساء والتوليد
- الجلدية
- الطب النفسي
- الغدد الصماء
- الجهاز الهضمي
- أمراض الدم والأورام
- طب الكلى
- طب القلب
- طب الرئة
- آخرون:

١٥. مقارنة بالزيارات في العيادات، كانت الزيارات الافتراضية: (أكثر فاعلية / فعالة بنفس القدر / أقل فعالية في) (اختر واحدة):

- توفير الوقت (أكثر فعالية / فعالية متساوية / أقل فعالية)
- توفير المال / النفقات (أكثر فعالية / فعالية متساوية / أقل فعالية)
- التحدث مع مقدم الرعاية الصحية الخاص بك (أكثر فعالية / فعالية متساوية / أقل فعالية)
- الرد على أسئلتك / استفساراتك (أكثر فعالية / فاعلية متساوية / أقل فعالية)
- تلبية احتياجاتي العامة من الرعاية الصحية (أكثر فعالية / بنفس الفعالية / أقل فعالية)
- الشعور بالأمان عند إجراء محادثة هاتفية أكثر من زيارة الطبيب لأن مرض COVID-19 (أكثر فعالية / بنفس الفعالية / أقل فعالية)
- احترام خصوصيتي وسريتي (أكثر فعالية / فعالية متساوية / أقل فعالية)

١٦. إذا تم اقتراح/ وصف دواء (أدوية)، فكيف حصلت عليهم؟

- استلام من صيدلية
- توصيل الأدوية إلى المنزل
- لم يتم وصف دواء لي

١٧.   هل ستستمر في استخدام المواعيد الافتراضية بعد انتهاء جائحة COVID-19؟ (نعم / لا / لست متأكدًا)

١٨. هل تنصح بزيارات افتراضية للآخرين؟ (نعم / لا / لست متأكدًا)

###

## Participant Information Sheet (English)

Project Information Sheet

The Prevalence and Perception of Telehealth Utilization: A Social Media Study

Invitation

You are being invited to take part in this research project. This study is being conducted by Dr. Khawla Ali, a Senior Clinical Lecturer at the Royal College of Surgeons in Ireland-Medical University of Bahrain. Before you decide to do so, it is important that you understand the objectives of this research project done and what it will involve. Please take time to read the following information carefully and discuss it with others if you wish. If you are unsure of any of the information provided or would like more information, feel free to contact us on [covidstudybh@rcsi.com](mailto:covidstudybh@rcsi.com). Take your time to decide whether or not you wish to take part.

Project Purpose

This research project aims to utilize an online, social media platform (Instagram) to find out the current usage of virtual methods (telephone calls, video calls, message chats) for visits with your health providers during the COVID-19 pandemic, compared to before the pandemic. And what you think about this method if you have used it.

Who Can Take Part in this Project?

Anyone who is 18 years and older.

Why Have You Been Chosen?

You have been chosen because you are an active social media user.

Do You Have to Take Part?

No, you do not have to. It is completely up to you to decide whether or not you would like to take part. You can withdraw from this survey at any time without giving a reason by not submitting it at the end. This way your information will not be saved.

What Do You Have to Do?

All that is asked of you is to answer our online survey that is made up of 17 questions about using "telehealth".

What are The Possible Risks of Taking Part?

Participating in this research project is not anticipated to cause you any disadvantages or discomfort. However, if you feel distressed or uncomfortable at any time please let us know at [covidstudybh@rcsi.com](mailto:covidstudybh@rcsi.com) and we will arrange for you to speak to a healthcare professional about this. This email is also provided to you at end of survey.

What are The Possible Benefits of Taking Part?

Whilst there are no immediate benefits for those people who choose to participate in this research project, it is hoped that this work will have a beneficial impact on how healthcare is delivered in the future.

Will My Answers Be Kept Confidential?

All your answers to the survey will be kept strictly confidential. You will not be identifiable in any reports nor publications. The data collected in the online survey will be stored online in a form protected by passwords and other relevant security processes and technologies.

If data is to be shared, it will be shared in an anonymized form, which will not allow any individuals to be identified.

What Will Happen to The Results of The Research Project?

Results of the research project will be analyzed and published in a medical journal. You will not be identified in any report or publication. If you wish to be given a copy of any reports resulting from this research project, please ask us to put you on our circulation list by contacting us on covidstudybh@rcsi.com. The PI will also be sharing the final results of everyone's responses on her platform after the study has been published.

Contact for Further Information

Dr Khawla Ali, consultant endocrinologist and senior lecturer at RCSI-MUB, Bahrain

Email: [covidstudybh@rcsi.com](mailto:covidstudybh@rcsi.com)

Thank you for taking the time to be a part of our research project.

### Participant Information (Arabic)

ورقة معلومات المشروع

الانتشار والتصور العام لاستخدام الخدمات الصحية عن بعد: دراسة وسائل التواصل الاجتماعي

رسالة دعوة

أنت مدعو للمشاركة في هذا المشروع البحثي. قبل أن تقرر القيام بذلك، من المهم أن تفهم أهداف مشروع البحث هذا وما سيتضمنه. البحث هذا يتم بإشراف الدكتورة خولة فؤاد، استاذة مساعدة في الكلية الملكية للجراحين في ايرلندا، جامعة البحرين الطبية. يرجى تخصيص بعض الوقت لقراءة المعلومات التالية بعناية ومناقشتها مع الآخرين إذا كنت ترغب في ذلك. إذا لم تكن متأكدًا من أي من المعلومات المقدمة أو كنت ترغب في مزيد من المعلومات، فلا تتردد في الاتصال ببريدنا الالكتروني. خذ وقتك لتقرر ما إذا كنت ترغب في المشاركة أم لا. شكرا لقراءتك.

[covidstudybh@rcsi.com](mailto:covidstudybh@rcsi.com)

الهدف من المشروع

يهدف هذا المشروع البحثي إلى استخدام منصة وسائل اجتماعية عبر الإنترنت (انستلقرام) تصل إلى أكثر من مئة و ثلاثون الف شخص يوميًا لإدارة استبيان يهدف إلى تقييم الانتشار الحالي لاستخدام الخدمات الصحية عن بُعد والمواقف العامة والاستعداد تجاه هذه الطريقة في تقديم الرعاية الصحية أثناء جائحة فيروس كورونا 2019 ، عبر بلدان مختلفة.

أهداف المشروع

هدفنا الأساسي من هذا المشروع البحثي هو معرفة مقدار عالمية استخدام الخدمات الصحية عن بُعد بين مستخدمي وسائل التواصل الاجتماعي أثناء و قبل جائحة فيروس كورونا 2019. تتمثل أهدافنا الثانوية في تحديد خصائص المستخدمين والتخصصات الطبية التي تم التواصل معها والاستعداد لاستخدام الخدمات الصحية عن بُعد والاستعداد للتوصية بالخدمات الصحية عن بُعد للآخرين.

##

من يمكنه المشاركة في هذا المشروع؟

أي شخص يبلغ من العمر 18 عامًا فما فوق.

لماذا تم اختيارك؟

لقد تم اختيارك لأنك مستخدم نشط لوسائل التواصل الاجتماعي وتجاوز عمرك 17 عامًا.

##

هل يجب عليك المشاركة؟

الأمر متروك لك تمامًا لتقرر ما إذا كنت ترغب في المشاركة أم لا. إذا قررت المشاركة في هذه الدراسة، يمكنك الانسحاب من هذا الاستطلاع في أي وقت دون إبداء الأسباب.

ماذا يجب عليك أن تفعل؟

كل ما يُطلب منك هو الإجابة على استبياننا عبر الإنترنت والمكون من 17 سؤالًا.

ما هي المخاطر المحتملة للمشاركة؟

من غير المتوقع أن تسبب لك المشاركة في هذا المشروع البحثي أي اضرار أو إزعاج. ستكون احتمالية حدوث أي ضرر جسدي و / أو نفسي هي نفسها التي قد تحدث في الحياة اليومية.

ما هي فوائد ممكنة من المشاركة؟

بينما لا توجد فوائد فورية لأولئك الأشخاص الذين يختارون المشاركة في هذا المشروع البحثي، فمن المأمول أن يكون لهذا العمل تأثير مفيد على كيفية تقديم الرعاية الصحية في المستقبل.

هل ستبقى إجاباتي سرية؟

سيتم الاحتفاظ بجميع إجاباتك على الاستبيان في سرية تامة. لن يتم التعرف عليك في أي تقارير أو منشورات. سيتم تخزين البيانات التي تم جمعها في الاستطلاع عبر الإنترنت في شكل محمي بكلمات مرور وعمليات وتقنيات الأمان الأخرى.

إذا كانت البيانات ستتم مشاركتها، فستتم مشاركتها في شكل مجهول الهوية، مما لن يسمح بتحديد هوية أي فرد.

ماذا سيحدث لنتائج المشروع البحثي؟

 سيتم تحليل نتائج المشروع البحثي ونشرها في مجلة طبية. لن يتم التعرف عليك في أي تقرير أو منشور. إذا كنت ترغب في الحصول على نسخة من أي تقارير ناتجة عن مشروع البحث هذا، فيرجى مطالبتنا بذلك عبر الاتصال بالبريد الإلكتروني الخاص بنا [covidstudybh@rcsi.com](mailto:covidstudybh@rcsi.com)

اتصل للحصول على مزيد من المعلومات

الرجاء التواصل مع الدكتورة خولة علي، استشارية الغدد الصماء واستاذة مساعدة في الكلية الملكية للجراحين في ايرلندا، جامعة البحرين الطبية

البريد الإلكتروني: **covidstudybh@rcsi.com**

*نشكرك على الوقت الذي استغرقته لتكون جزءًا من مشروعنا البحثي.*

## Participant Consent Form (English)

RCSI Bahrain study participant consent form

The Prevalence and Perception of Telehealth Utilization: A Social Media Study

I have read and understood the Patient Information Sheet about this research project. The information has been fully clear to me and I have been able to ask questions via the email provided, all of which have been answered to my satisfaction. I understand that I don't have to take part in this study and that I can opt out at any time. I understand that I don't have to give a reason for opting out and I understand that opting out won't affect my future. I give permission for my anonymous data to be stored. I give permission for publication of anonymised results.

By proceeding with this survey, I agree, consent, and concur with all the above.

### Participant Consent Form (Arabic)

نموذج موافقة المشارك في الدراسة في الكلية الملكية للجراحين في البحرين

الانتشار والتصور العام لاستخدام الخدمات الصحية عن بعد: دراسة وسائل التواصل الاجتماعي

لقد قرأت وفهمت معلومات المشروع حول هذا المشروع البحثي. تم شرح المعلومات بالكامل لي وتمكنت من طرح الأسئلة، وقد تم الرد عليها جميعًا بما يرضي. افهم انه لا يتعين علي المشاركة في هذه الدراسة وأنه يمكنني إلغاء الاشتراك في أي وقت. أفهم أنه لا يتعين علي تقديم سبب لإلغاء الاشتراك وأدرك أن الانسحاب لن يؤثر على رعايتي المستقبلية. انا على دراية بالمخاطر المحتملة لهذه الدراسة البحثية. أعطي الإذن لتخزين البيانات السرية ونشرها بمجلة علمية.

مواصلتي بإكمال الاستبيان هو إقرار مني على موافقتي بكل ما ذكر أعلاه
